# Adrenal Hematoma Volume as a Predictor of Morbidity and Mortality in Traumatic Adrenal Injury

**DOI:** 10.3390/jcm14155566

**Published:** 2025-08-07

**Authors:** Yiğit Türk, Aykut Özkılıç, Hüsnügül Karakoç, Recep Temel, Ezgi Güler, Gökhan İçöz, Özer Makay, Murat Özdemir

**Affiliations:** 1 Department of General Surgery, Division of Endocrine Surgery, Ege University Hospital, Izmir 35100, Turkey; yigitturk87@gmail.com (Y.T.); aykutozkilic.16@gmail.com (A.Ö.); doktorreceptemel@gmail.com (R.T.); icozgokhan@gmail.com (G.İ.); 2 Department of Radiology, Ege University Hospital, Izmir 35100, Turkey; gulkarakoc35@gmail.com (H.K.); gulerezgi@yahoo.com (E.G.); 3 Center for Endocrine Surgery, Ozel Saglik Hospital, Izmir 35000, Turkey; makayozer@yahoo.com; 4 School of Medicine, Aristotle University, 54124 Thessaloniki, Greece; 5 Instituto Português De Oncologia De Coimbra Francisco Gentil, 3000075 Coimbra, Portugal

**Keywords:** traumatic adrenal injury, adrenal hematoma, abdominal trauma, blunt trauma

## Abstract

**Background**: Traumatic adrenal injury (TAI) is a rare but significant condition that affects 2.5% of patients with thoracoabdominal trauma. The impact of adrenal hematoma volume on clinical outcomes remains underexplored. This study aimed to evaluate factors associated with morbidity and mortality in patients with TAI, with a particular focus on adrenal hematoma volume as a predictive marker. **Methods**: Retrospective data from patients with radiologically confirmed TAI between 2013 and 2023 was analyzed. Clinical, demographic, and radiological variables were reviewed. Hematoma volume was calculated from computed tomography (CT) imaging and analyzed using univariate and multivariate models. Receiver operating characteristic (ROC) analysis was employed to evaluate its predictive accuracy. **Results**: Sixty patients were included in the study. The median hematoma volume was 16.0 cm^3^, with a predominance of injuries on the right side. The morbidity and mortality rates were 18.3% and 8.3%, respectively. Univariate analysis identified a lower Glasgow Coma Scale (GCS) score, higher Injury Severity Score (ISS), and increased hematoma volume as significant factors. In multivariate analysis, hematoma volume and GCS score remained independent predictors of adverse outcomes. A volume threshold of >23 cm^3^ was associated with significantly higher morbidity and mortality (AUC = 0.80, 95% CI: 0.68–0.92). **Conclusions**: This study is the first to demonstrate that the volume of adrenal hematoma is an independent predictor of adverse outcomes in patients with traumatic adrenal injury. Integrating volume into clinical assessment may help identify high-risk patients requiring improved observation and management.

## 1. Introduction

Traumatic adrenal injury (TAI) is an uncommon occurrence, observed in approximately 2.5% of blunt thoracoabdominal trauma cases [1]. It is frequently associated with injuries to other intra-abdominal or thoracic organs, with the associated morbidity and mortality generally determined by the extent and severity of these concurrent injuries [2,3]. TAI is generally managed conservatively; however, invasive interventions, including angioembolization or surgery, may be necessary in cases of active hemorrhage, hemodynamic instability, or progressive hematoma expansion. Surgery is primarily conducted for injuries to other organs rather than the adrenal glands specifically [4,5].

The Injury Severity Score (ISS) was established as an accepted method for assessing the severity of trauma [6]. The ISS, Glasgow Coma Scale (GCS) score, hemodynamic status, and concurrent organ injuries have been associated with poorer outcomes in patients with TAI. The American Trauma Association suggested an imaging-based classification system for adrenal injury; however, its clinical application remains restricted, and research evaluating its efficacy in the literature is limited [7].

The hypothalamic–pituitary–adrenal (HPA) axis is crucial for maintaining physiological homeostasis, especially during trauma or critical illness [8,9]. However, injury to the adrenal glands, as seen in cases of TAI, may disrupt this delicate balance, leading to worse clinical outcomes and increased management complexity. In this situation, the early identification of high-risk patients is essential to guide treatment modalities and improve outcomes. This study investigates factors related to morbidity and mortality following TAI, with a particular focus on adrenal hematoma volume. To the best of our knowledge, this is the first study to investigate the volume of adrenal hematomas as a possible predictor of prognosis.

## 2. Materials and Methods

We retrospectively reviewed the radiological data of patients aged 18 years and older who were admitted to Ege University Hospital with thoracoabdominal trauma between January 2013 and December 2023. Patient computed tomography (CT) images were obtained from the hospital’s Picture Archiving and Communication System (PACS) (IDS7, Sectra, Linköping, Sweden). All CT images were re-evaluated by a radiologist with five years of experience in abdominal imaging and a radiology resident using a consensus-based approach. Volumetric measurements of adrenal hematomas were performed by a single investigator under the supervision of the same radiologist.

Only patients with radiologically confirmed trauma-related adrenal injuries were included in the study, while those with adrenal masses, metastatic lesions, or no documented history of trauma were excluded.

The following variables were collected and analyzed: age, sex, comorbidities, mechanism and side of injury, the presence of associated organ injuries, and adrenal hematoma volume. The GCS score, ISS, heart rate (HR), mean arterial pressure (MAP), and plasma glucose levels were analyzed upon admission to the emergency department. The analysis included changes in lesion size, duration of hospital stay, blood product transfusions, and surgical interventions during follow-up.

**Figure 1:**
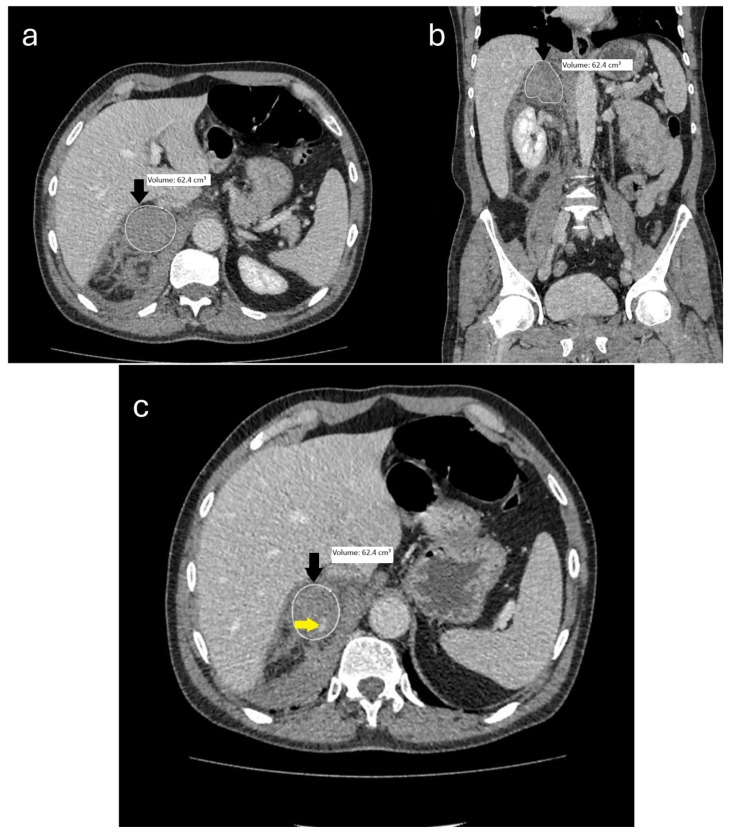
A 63-year-old male patient was admitted to the emergency department following a motor vehicle accident. Contrast-enhanced axial (**a**,**c**) and coronal (**b**) abdominal CT images demonstrate a hematoma in the right adrenal gland (black arrows), with associated hemorrhagic fluid extending into the right periadrenal and perirenal spaces, as well as Morrison’s pouch. On the portal venous phase CT (**c**), the yellow arrow indicates contrast extravasation within the adrenal hematoma, suggestive of active bleeding.

Due to the retrospective design of the study, CT scans obtained over a 10-year period were included. All images were reviewed to ensure adequate diagnostic quality prior to analysis. Although some variation in CT hardware and software may have occurred during this period, all volumetric assessments were performed using standardized segmentation protocols and consistent measurement criteria to minimize variability. Adrenal hematoma volume was manually measured on axial CT images with a slice thickness of 1.25 mm. All scans were acquired in the portal venous phase using the institutional PACS, and volumetric measurements were conducted with Sectra 3D software (Version 26.2.15.8535. Sectra AB, Linköping, Sweden) (Figure 1).

Patients were categorized into three groups based on their clinical outcomes, namely vital, morbid, or mortal. The vital group included patients who did not develop any systemic or organ-specific complications. The morbid group comprised those who experienced clinically significant complications, such as pneumonia, sepsis, pulmonary embolism, surgical site infection, or bile leakage. The mortal group consisted of patients who died in the hospital due to trauma-related causes. For the purpose of multivariate analysis, patients with either morbidity or mortality were combined under the category of adverse clinical outcomes.

In addition, patient records were reviewed for evidence of adrenal insufficiency and the need for hormone replacement therapy during a follow-up period of at least one year.

The Shapiro–Wilk test was applied to evaluate the normality of the data. Non-normally distributed continuous data were evaluated with the Mann–Whitney U test, while categorical variables were analyzed using the chi-squared test or Fisher’s exact test. Binary logistic regression analysis was performed to determine the factors associated with morbidity and mortality. Receiver operating characteristic (ROC) analysis was performed to assess the predictive value of adrenal hematoma volume in relation to morbidity and mortality. A *p*-value of less than 0.05 was deemed significant. Results are reported with valid 95% confidence intervals (CIs). All statistical analyses were conducted using SPSS (IBM SPSS Statistics 25.0) and Python (Version 3.11, Statsmodels, Scipy).

## 3. Results

This study initially included 69 patients in total. Following a radiological reassessment, seven patients were excluded due to an absence of adrenal injury and two due to adrenal hematoma associated with adrenal masses and no history of trauma. Thus, the final analysis included 60 patients diagnosed with traumatic adrenal injury. The mean age of the patients was 46.2 ± 17.2 years, and 80% (*n* = 48) of the patients were male. Comorbidities were identified in 18.4% (*n* = 11) of the patients, whereas 11.7% (*n* = 7) were receiving anticoagulant therapy (Table 1).

Motor vehicle collisions (MCVs) accounted for the majority of injuries (55%), followed by falls from a height (33.3%), bicycle accidents (6.7%), and other mechanisms (5%). Right-sided adrenal injuries were most common (81.7%), while left-sided injuries accounted for 15% and bilateral injuries for 3.3%. Thoracic trauma, which includes rib fractures, pneumothorax, hemothorax, or pulmonary contusion, was detected in 75% of patients. Other injuries were distributed as the following percentages: liver (53.3%), spinal (45%), pelvis (18.3%), extremities (16.7%), kidney (15%), spleen (11.7%), and cranial (10%). Isolated duodenal injury occurred in 3.3% of cases. Isolated adrenal gland injury was observed in 5% of patients. Contrast extravasation on CT was identified in 8.3%. The median hematoma diameter was 32.5 mm [IQR: 31.45–41.98], and the median hematoma volume was 16.0 cm^3^ [range: 1.0–135.0] (Table 1).

Follow-up imaging was obtained for 78.3% of patients, with a mean interval of four days. In this cohort, hematoma size was stable in 68% of cases, regressed in 25%, and progressed in 6.4%. Conservative management was utilized in 65% of patients, whereas 35% necessitated surgical intervention for associated injuries. One patient (1.7%) underwent angioembolization. No patients required or underwent adrenalectomy. The median length of hospital stay (LOS) was 7 days (range 1–152 days).

The morbidity rate was 18.3% (*n* = 11), with the most common complication being pneumonia (13.3%), followed by sepsis (5%), pulmonary embolism (5%), surgical site infection (5%), and bile leakage after liver surgery (1.7%). Mortality was observed in 8.3% (*n* = 5) of patients. Two patients died from septic shock and respiratory failure following the surgical repair of trauma-related duodenal injuries. Two patients died from hemorrhagic shock after laparotomy. One patient died from brain death caused by severe cranial trauma.

There was no significant correlation between follow-up imaging findings (e.g., hematoma stability, regression, or progression) and clinical outcomes (*p* = 0.62). Of the patients with radiological progression, only one required angioembolization. Patients with complications had more extended hospital stays (*p* < 0.01) (Table 2).

A comparative analysis revealed no statistically significant associations between clinical outcomes and variables, including age, sex, or concurrent medical conditions. Anticoagulant use (*p* = 0.02) and the occurrence of left-sided and bilateral adrenal injuries (*p* = 0.02) were related to worse outcomes. Morbidity and mortality exhibited a significant association with concurrent splenic (*p* < 0.01) and duodenal injuries (*p* < 0.01).

Univariate analysis revealed significant differences between outcome groups for the GCS score (*p* < 0.001), ISS (*p* = 0.001), and MAP (*p* = 0.01). The findings strongly suggest that each parameter significantly contributes to our ability to predict clinical deterioration, highlighting their importance in improving patient outcomes. Multivariate analysis showed that a lower GCS score was an independent predictor of increased morbidity and mortality (HR: 0.294; 95% CI: 0.104–0.832; *p* = 0.02). The ISS showed a limited association with worse outcomes (HR: 1.180; 95% CI: 0.996–1.398; *p* = 0.05), while the MAP did not (HR: 1.093; 95% CI: 0.972–1.229; *p* = 0.15). Increased adrenal hematoma volume was identified as an independent predictor of adverse outcomes (HR: 1.152; 95% CI: 1.025–1.295; *p* = 0.01). No significant multicollinearity was detected (all VIFs < 2.5). (Table 3).

An ROC analysis was conducted to assess the predictive value of adrenal hematoma volume concerning adverse clinical outcomes. The area under the curve (AUC) was 0.803 (95% CI: 0.681–0.924). Using the Youden index, an optimal cut-off value of 23.0 cm^3^ was identified. Patients with hematoma volumes exceeding this threshold had higher rates of complications and mortality. At this cut-off, the sensitivity was calculated as 62.5%, while the specificity was 81.8%.

To explore this further, patients were stratified into two groups, namely those with hematoma volume ≤ 23 cm^3^ and those with > 23 cm^3^. Univariate analysis revealed significant differences in hematoma laterality (*p* = 0.03) and the incidence of complications (*p* < 0.01) between the two volume groups (Table 4).

During a minimum one-year follow-up period, none of the surviving patients showed clinical evidence of adrenal insufficiency or required hormonal replacement therapy.

## 4. Discussion

The incidence of TAI in patients with thoracoabdominal trauma ranges from 0.44% to 2.5%, consistent with our findings and the previous literature [1,3,10]. The mean patient age in our cohort was 46.2 years, in line with previous research, including that of Digiacomo et al. [10]. Right-sided adrenal injuries were more common, a trend explained by anatomical factors including the shorter venous drainage of the right adrenal gland into the inferior vena cava and its position between the liver and vertebrae [1,11]. Bilateral injuries were rare in our cohort (3.3%), considerably lower than the 38% reported by Panda et al. [12]. This dissimilarity may be attributed to variations in imaging techniques or patient selection. Consistent with previous research, bilateral and left-sided adrenal injuries in our study were associated with increased morbidity and mortality, likely reflecting greater trauma severity [12]. Thoracic injuries were the most common associated trauma, affecting approximately 75% of patients. Intra-abdominal solid organ injury was also common, particularly to the liver, kidney, and spleen, which is consistent with previous studies [2].

The median hematoma diameter in this study was 32.5 mm, with a median volume of 16.0 cm^3^, consistent with previous studies [2,13]. Multivariate analysis confirmed hematoma volume as an independent predictor of adverse outcomes (HR: 1.15, 95% CI: 1.03–1.29; *p* = 0.01), implying its potential practicality in clinical risk stratification and management decisions. Considering its prognostic importance, we conducted a detailed assessment of adrenal hematoma volume through ROC analysis. The AUC was 0.80, showing strong discriminatory ability. A cut-off value of 23.0 cm^3^ was established, indicating that patients exceeding this threshold experienced significantly higher rates of complications and mortality, with a sensitivity of 62.5% and specificity of 81.8%. This threshold may serve as a practical approach for initial clinical risk stratification.

The American Association for the Surgery of Trauma (AAST) has developed a classification system for traumatic adrenal injury [7]. Nguyen et al. categorized traumatic adrenal injury into low and high grades based on the AAST criteria, observing no significant difference in patient outcomes between these classifications [14]. The AAST grading system for adrenal injury, though commonly referenced, has limited clinical utility due to the small size and anatomical complexity of the adrenal gland. Accordingly, we did not apply this system in our study, opting instead for a volume-based assessment.

Our findings demonstrate for the first time that adrenal hematoma volume is a significant prognostic marker in TAI. A threshold of 23.0 cm^3^ was associated with increased morbidity and mortality, underscoring its potential value in early risk stratification. Nevertheless, the single-center design may limit the generalizability of our findings, particularly across institutions with varying trauma systems and management protocols. Differences in trauma mechanisms, imaging techniques, and institutional practices across healthcare settings may further affect their broader applicability.

To enhance external validity and clinical integration, these findings require validation in larger, multicenter cohorts. The practical implementation of this volume threshold in real-time settings depends on the availability of rapid and reproducible volumetric assessment. Emerging automated or semi-automated tools, including those utilizing artificial intelligence, may facilitate efficient and standardized volume estimation in emergency care.

In our study, several clinical factors—including the GCS score, ISS, MAP, blood glucose, need for transfusion, and surgical procedures—were linked to worse outcomes in univariate analysis. However, when all variables were evaluated together, only hematoma volume and the GCS score remained as independent predictors of morbidity and mortality. Prior research has established a relationship between the ISS and mortality [2,5,15], whereas Digiacomo et al. found no such correlation [16]. Our study indicates that the ISS approached statistical significance in multivariate analysis (HR: 1.18, 95% CI: 1.00–1.40; *p* = 0.05). This suggests that its prognostic value may become clearer in studies with larger sample sizes. Due to the polytrauma nature of many patients, it is possible that increased hematoma volume indicates more severe injury mechanisms. However, the persistence of hematoma volume as an independent predictor in the multivariate analysis indicates that it is relevant beyond overall injury severity.

Pneumonia was the most frequently observed complication, followed by sepsis, consistent with prior research [2]. Patients in the morbidity group encountered a longer duration of hospital stay. The mortality rate identified in this study was 8.3%, consistent with the rate reported by Liao et al. [5]. Previous studies have reported mortality rates from 10% to 32.9% [2,15,17]. Mortality rates have decreased over time. This trend may suggest improvements in trauma care, advances in critical care and imaging, and a more selective approach to surgical intervention, which may lead to a reduction in unnecessary procedures in patients with TAI.

The management of traumatic adrenal injury (TAI) depends primarily on the existence of concomitant intra-abdominal injuries and the hemodynamic stability of the patient. Adrenal injuries are typically managed through conservative approaches [3]. Liao et al. reported one adrenalectomy, five cases managed with adrenal sutures and hemostasis, and four cases treated with angioembolization in their case series [5]. Stawicki et al. reported adrenalectomy and nephrectomy rates of 2.5% and 4.3%, respectively [2]. Alyasali et al. reported that while 25% of patients with intra-abdominal trauma underwent laparotomy, none necessitated adrenalectomy or nephrectomy [1]. Twelve patients (20%) in our study underwent laparotomy as a result of abdominal injuries. Seven of the patients underwent surgical exploration and hemostasis, two had splenectomies, two required duodenal repair, and one underwent hepatectomy. Furthermore, none required adrenalectomy or nephrectomy. One patient underwent angioembolization for an enlarging adrenal hematoma.

Adrenal insufficiency, a known complication of TAI, was not clinically observed in any surviving patients during the one-year follow-up. However, ACTH and cortisol levels were not routinely measured. Thus, subclinical or delayed-onset adrenal dysfunction cannot be ruled out. This observation is consistent with recent reports indicating a low incidence of clinically evident adrenal insufficiency after trauma [1,18]. Prolonged monitoring is essential, especially in cases of bilateral adrenal injury, due to the risk of subclinical or delayed-onset adrenal dysfunction. Periodic hormonal evaluation in these patients may facilitate the early identification and management of potential endocrine complications [19,20].

This study has several limitations. Although all thoracoabdominal trauma cases were re-evaluated, the retrospective design introduces an inherent risk of selection bias. Traumatic adrenal injuries that were clinically silent or radiologically subtle may have gone undetected, which could have skewed the cohort toward more severe cases with larger hematomas. Adrenal insufficiency did not occur based on clinical observation during follow-up. However, no biochemical evaluation of adrenal function was performed, and ACTH and cortisol levels were not measured. Finally, the single-center setting may limit the generalizability of our findings. Further prospective, multicenter studies are needed to validate the clinical utility of adrenal hematoma volume in risk stratification.

## 5. Conclusions

This study establishes for the first time that the volume of adrenal hematoma is an independent predictor of morbidity and mortality in patients with traumatic adrenal injury. Identifying a specific volume threshold linked to adverse outcomes offers a practical and objective approach for early clinical risk assessment.

## Figures and Tables

**Table 1:** Demographic variable and trauma mechanism.

Variable	*n*	%
Age	46.2 ± 17.2	
Gender		
Males	48	80
Females	12	20
Comorbidities		
Cardiovascular Disease	7	11.70
Diabetes Mellitus	2	3.30
Cerebrovascular Disease	1	1.70
Respiratory System Diseases	1	1.70
Anticoagulant/Antiaggregant Use	7	11.70
Mechanism of Trauma		
MVC-Related	33	55
Fall from Height	20	33.30
Bicycle Accident	4	6.70
Other	3	5
Adrenal Hematoma Side		
Right	49	81.70
Left	9	15
Bilaterally	2	3.30
Associated Injuries		
Thorax	45	75
Liver	32	53.30
Spinal	27	45
Pelvic	11	18.30
Cranial	10	10
Extremity	10	16.70
Kidney	9	15
Splenic	7	12
Duodenum	2	3
Isolated Adrenal Gland Injury	3	5
Contrast Extravasation	5	8
Diameter of Hematoma (median, mm)	32.5 [27–40]	
Volume of Hematoma (median, cm^3^)	16.0 [1.0–135.0]	

MVC: Motor vehicle accident.

**Table 2:** Univariate analysis of variables influencing morbidity and mortality outcomes.

Variable	Vital (*n* = 44)	Morbid (*n* = 11)	Mortal (*n* = 5)	*p* Values
Age	44.93 ± 15.52	46 ± 19.14	57.8 ± 25.9	0.28
Gender				
Male	34 (77.3%)	9 (81.8%)	5 (100%)	
Female	10 (22.7%)	2 (18.2%)	0	0.26
Comorbidities				
Yes	5 (11.4%)	2 (18.2%)	2 (40%)	
No	39 (86.6%)	9 (81.8%)	3 (60%)	0.10
Anticoagulant use				
Yes	3 (6.8%)	2 (18.2%)	2 (40%)	
No	41 (93.2%)	9 (81.8%)	3 (60%)	**0.02**
Adrenal hematoma side				
Right	38 (86.4%)	8 (72.7%)	3 (60%)	
Left	6 (13.6%)	2 (18.2%)	1 (20%)	
Bilaterally	0	1 (9.1%)	1 (20%)	**0.02**
Median adrenal hematoma volume (cm^3^)	13.5 [1.0–62.0]	30.0 [10.0–110.0]	18.0 [12.0–135.0]	**0.001**
Contrast extravasation	3 (6.8%)	2 (18.2%)	0	0.85
Associated injuries				
Thorax	32 (72.7%)	10 (90.9%)	3 (60%)	0.90
Liver	23 (52.3%)	6 (54.5%)	3 (60%)	0.74
Spine	17 (38.6%)	6 (54.7%)	4 (80%)	0.06
Pelvic	8 (18.2%)	2 (18.2%)	1 (20%)	0.93
Cranial	7 (15.9%)	3 (27.3%)	0	0.78
Extremity	8 (18.2%)	2 (18.2%)	0	
Kidney	8 (18.2%)	0	1 (20%)	0.51
Splenic	3 (66.8%)	1 (9.1%)	3 (60%)	**<0.01**
Duodenum	0	0	2 (40%)	**0.001**
GCS (median,range)	15 (11–15)	12 (4–14)	6 (5–12)	**<0.001**
ISS (median,range)	14 (4–38)	33 (6–43)	27 (18–66)	**0.001**
HR, bpm (median,range)	90 (63–140)	103 (67–140)	127 (96–164)	0.05
MAP, mmHg (median,range)	89 (62–119)	76 (46–116)	78 (45–116)	**0.01**
P-glucose, mg/dL(median,range)	123 (90–368)	146 (99–298)	190 (161–294)	**0.01**
Received blood transfusion	11 (25%)	9 (81.8%)	4 (80%)	**<0.001**
Angioembolization	0	1 (%100)	0	0.10
Any operation	9 (20.5%)	8 (72.7%)	5 (100%)	**<0.001**
Operation type				
Exploratory laparotomy	1 (2.3%)	3 (27.3%)	3 (60%)	**<0.001**
Splenectomy	1 (2.3%)	1 (9.1%)	0	0.73
Pelvic/spinal surgery	4 (9.1%)	2 (18.2%)	0	0.94
Extremity surgery	3 (6.8%)	1 (9.1%)	0	0.74
Hepatectomy	0	1 (9.1%)	0	0.30
Duodenal surgery	0	0	2 (40%)	**<0.001**
Control imaging				
Stabil	25 (78.1%)	6 (18.8%)	1 (3.1%)	
Regression	8 (66.7%)	4 (33.3%)	0	
Progressive	2 (66.7%)	1 (33.3%)	0	0.62
LOS (day)	6.5 [6.14–9.18]	32 [16.84–79.15]	3 [0.71–4.48]	**<0.001**

GCS: Glasgow Coma Scale; ISS: Injury Severity Score; HR: heart rate; MAP: mean arterial pressure; BPM: beats per minute; P-glucose: plasma glucose; LOS: length of stay. Bold values are statistically significant.

**Table 3:** Multivariate analysis of the factors influencing patient morbidity and mortality.

Variable	HR	95.0% CI	*p*-Value
GCS	0.29	[0.11–0.81]	**0.01**
ISS	1.18	[1.00–1.40]	0.05
MAP (mmHg)	1.09	[0.97–1.23]	0.15
Hematoma volume (cm^3^)	1.15	[1.03–1.29]	**0.01**

GCS: Glasgow Coma Scale; ISS: Injury Severity Score; MAP: mean arterial pressure.

**Table 4:** Comparison of clinical and demographic characteristics based on adrenal hematoma volume (≤23 cm^3^ vs. >23 cm^3^) in patients with traumatic adrenal injury.

Variable	Adrenal Hematoma ≤ 23 cm^3^ (*n* = 34)	Adrenal Hematoma > 23 cm^3^ (*n* = 26)	*p* Value
Adrenal hematoma side			
Right	37 (75.5%)	12 (24.5%)	
Left	8 (88.9%)	1 (11.1%)	
Bilaterally	0	2 (100%)	**0.03**
Contrast extravasation	2 (40.0%)	3 (60.0%)	0.09
GCS	15 [14.0–15.0]	13 [11.5–15.0]	0.07
ISS	17.0 [12.0–27.0]	17.0 [12.5–30.0]	0.62
HR, bpm	95 [80.0–105.0]	103 [74.5–120.5]	0.45
MAP, mmHg	86.0 [80.0–94.0]	80.0 [71.5–94.5]	0.42
Received blood transfusion	16 (35.6%)	8 (53.3%)	0.24
Morbidity	4 (36.4%)	7 (63.6%)	**0.003**
Pneumonia	2 (25.0%)	6 (75.0%)	**0.002**
Sepsis	1 (33.3%)	2 (66.7%)	0.15
Pulmonary embolism	2 (66.7%)	1 (33.3%)	1.00
Biliary leak	0	1 (100%)	0.25
Surgical site infection	1 (33.3%)	2 (66.7%)	0.15
Control imaging			
Stabil	18 (56.25%)	14 (43.75%)	
Regression	7 (58.3%)	5 (41.6%)	
Progressive	1 (33.3%)	2 (66.6%)	0.72
Mortality	2 (2.9%)	3 (5.8%)	0.64
LOS (day,median,IQR)	7 [5.0–9.0]	10 [5.5–34.0]	0.22

GCS: Glasgow Coma Scale; ISS: Injury Severity Score; HR: heart rate; MAP: mean arterial pressure; LOS: length of stay.

## Data Availability

The data presented in this study are available upon request from the corresponding author due to ethical restrictions.
